# An Update on CARD Only Proteins (COPs) and PYD Only Proteins (POPs) as Inflammasome Regulators

**DOI:** 10.3390/ijms21186901

**Published:** 2020-09-20

**Authors:** Savita Devi, Christian Stehlik, Andrea Dorfleutner

**Affiliations:** 1 Department of Pathology and Laboratory Medicine, Cedars Sinai, Los Angeles, CA 90048, USA; Fnu.SavitaDevi@cshs.org; 2 Department of Biomedical Sciences, and Samuel Oschin Comprehensive Cancer Institute, Cedars Sinai, Los Angeles, CA 90048, USA; 3 Department of Biomedical Sciences, Cedars Sinai, Los Angeles, CA 90048, USA

**Keywords:** AIM2 like receptor, ALR, ASC, caspase-1, CARD, caspase recruitment domain, COP, POP, PYD, PYRIN domain, inflammasome, interleukin-1, NLR, Nod-like receptor

## Abstract

Inflammasomes are protein scaffolds required for the activation of caspase-1 and the subsequent release of interleukin (IL)-1β, IL-18, and danger signals, as well as the induction of pyroptotic cell death to restore homeostasis following infection and sterile tissue damage. However, excessive inflammasome activation also causes detrimental inflammatory disease. Therefore, extensive control mechanisms are necessary to prevent improper inflammasome responses and inflammatory disease. Inflammasomes are assembled by sequential nucleated polymerization of Pyrin domain (PYD) and caspase recruitment domain (CARD)-containing inflammasome components. Once polymerization is nucleated, this process proceeds in a self-perpetuating manner and represents a point of no return. Therefore, regulation of this key step is crucial for a controlled inflammasome response. Here, we provide an update on two single domain protein families containing either a PYD or a CARD, the PYD-only proteins (POPs) and CARD-only proteins (COPs), respectively. Their structure allows them to occupy and block access to key protein–protein interaction domains necessary for inflammasome assembly, thereby regulating the threshold of these nucleated polymerization events, and consequently, the inflammatory host response.

## 1. Introduction

Inflammasomes represent a central component of the innate immune system and their discovery in 2002 started a tremendous effort to delineate the molecular mechanisms of inflammasome signaling and the downstream consequences [1]. Inflammasomes are multimeric protein scaffolds, comprised of an upstream cytoplasmic pattern recognition receptor (PRR), an adaptor and a downstream effector, which are assembled upon the sensing of pathogen associated molecular patterns (PAMPs) or damage associated molecular pattern (DAMPs). Recruitment of the adaptor to the activated PRR allows effector recruitment, followed by its activation and substrate cleavage. The effector caspase-1 cleaves interleukin (IL)-1β, IL-18, and gasdermin D (GSDMD), resulting in cytokine release and the induction of pyroptotic cell death to eliminate infections and restore homeostasis [1,2,3,4].

## 2. Inflammasome Sensors

Several cytosolic PRRs have been identified as sensors for canonical inflammasomes and the best studied are Nod like receptor (NLR) family pyrin domain (PYD)-containing 1 (NLRP1), NLRP3, neuronal apoptosis inhibitory proteins (NAIPs)/NLR caspase recruitment domain (CARD) containing 4 (NLRC4), absent in melanoma 2 (AIM2), and Pyrin [1,5,6,7,8,9,10,11,12,13,14,15,16,17,18,19,20,21]. NLRP3 consists of an N-terminal PYD for adaptor protein binding, a central NACHT domain containing NTPase activity involved in oligomerization and regulatory C-terminal leucine rich repeats (LRRs). NLRP3 responds to heterogeneous stimuli, including crystalline substances, microbial components, nucleic acids, extracellular ATP, membrane damaging toxins, membrane pore formation, organelle damage and ion imbalance [22,23,24]. The unifying mechanism is still elusive, but all stimuli trigger cellular stress resulting in K^+^, Cl^−^, Ca^++^ ion fluxes, mitochondrial, lysosomal, and trans Golgi network disruption, and metabolic changes [24]. Therefore, NLRP3 has a central role by sensing cellular perturbations during sterile inflammation and infection. NLRP1 was the first described inflammasome, and while human NLRP1 consists of a function to find domain (FIIND) and a C-terminal CARD in addition to the domains present in NLRP3, the three mouse paralogous genes lack the PYD [1]. NLRP1 is unique, as it is activated by a functional degradation mechanism and is therefore a sensor for pathogen activity, rather than sensing a specific PAMP. Activation occurs by the N-end rule degradation pathway, which removes the autoinhibitory N-terminal domain [18,19,25]. Autoproteolysis at the FIIND prevents degradation of the C-terminal FIIND-CARD fragment, which then functions as a caspase-1-activating scaffold [25,26,27,28]. N-end rule degradation is triggered by the *Bacillus anthracis* endopeptidase anthrax lethal toxin or by the *Shigella flexneri* E3 ubiquitin ligase ipaH7.8 [18,19,29]. The NAIP-NLRC4 inflammasome is activated by bacterial flagellin and components of the bacterial type III secretion system (T3SS) [9,10,11,20,21,30,31,32]. However, NLRC4 is not the direct sensor, but partners with upstream NAIP proteins, which determine the bacterial ligand specificity of the NLRC4 inflammasome [20,21,33,34,35]. While mice encode seven NAIP proteins, with NAIP1 and NAIP2 detecting the T3SS rod and needle proteins, respectively and NAIP5 and NAIP6 collectively detecting flagellin by their NACHT, humans encode only a single NAIP protein recognizing both the T3SS needle protein and flagellin [20,21,33,34,35,36,37,38,39,40]. AIM2 has a distinct domain architecture from NLRs and functions as a universal, cytosolic dsDNA sensor [12,13,14,15]. It consists of an N-terminal PYD and a C-terminal HIN-200 domain and detects cytosolic dsDNA in a sequence independent, but length dependent manner through the HIN-200 domain [41,42,43]. Comparable to NLRs, AIM2 also exists in an autoinhibited conformation in naïve cells, where the PYD blocks the HIN-200 domain, and this autoinhibition is released by interaction of the HIN-200 domain with dsDNA by electrostatic interaction [15]. During homeostasis, DNA is absent from the eukaryotic cytoplasm but if DNA is present, it acts as a danger signal to alert the innate immune system. However, DNA might accumulate inside the cytosol following nuclear membrane damage or following ionizing radiation exposure, which also leads to AIM2 activation [44,45]. Pyrin is the other guard-type sensor, which detects pathogen activity or cell damage as a consequence of toxin-mediated impairment of the RhoA GTPase activity. Also, cellular defects in an upstream pathway, such as mutations, that inevitably prevent RhoA GTPase activity, can lead to Pyrin inflammasome activation [17,46,47]. Pyrin also has a distinct domain architecture compared to NLRs, consisting of a PYD, a B-box type zinc finger domain, a coiled-coil domain and a B30.2/SPRY domain. RhoA activity enables phosphorylation of Pyrin by PKN1 and PKN2 on Ser208 and Ser242, which maintains an inactive conformation by binding to 14-3-3 chaperons [47,48,49,50]. Hence, the loss of 14-3-3 binding results in Pyrin inflammasome activation. Pyrin may also detect perturbations of the actin cytoskeleton, as mutations in PSTPIP1, a Pyrin binding partner, WDR1, and ARPC1B that affect actin polymerization, are detected by the pyrin inflammasome [51,52].

Several other, but less well characterized, PRRs have also been implicated in the formation of an inflammasome, including NLRP2, NLRP6, NLRP7, NLRP9b, NLRP12, NLRC5, and IFI16 [53,54,55,56,57,58,59,60,61]. NLRP2 forms an ATP-sensing inflammasome in astrocytes and neurons and contributes to inflammation in the central nervous system [53,54]. NLRP6 regulates an inflammasome in colonic epithelial cells controlling IL-18-mediated intestinal homeostasis by sensing lipoteichoic acid (LTA) of Gram-positive bacterial membranes [55,56]. NLRP7, which is restricted to humans, senses acylated lipopeptides present primarily in Gram-positive bacteria and *Mycoplasma* spp. [57,62,63]. Nlrp9b assembles an inflammasome in colonic epithelial cells and restricts rotavirus infection by sensing short dsRNA in concert with the Dhx9 helicase [58]. NLRP12 senses *Yersinia pestis* tetra-acylated lipid A, but also regulates NF-κB and MAPK activation and intestinal homeostasis [59,64,65,66]. The involvement of NLRC5 in inflammasome activation is still very poorly characterized but it has been suggested to cooperate with NLRP3 in sensing several bacteria [60]. IFI16 is unique, as it may sense the Kaposi sarcoma-associated herpesvirus (KSHV) genome and assembles a nuclear inflammasome in endothelial cells [61].

In addition to the above described canonical inflammasomes, which result in caspase-1 activation, a non-canonical inflammasome results in the activation of human caspase-4 and caspase-5 as well as mouse caspase-11 in response to detecting intracellular lipopolysaccharide (LPS) [67,68,69,70,71,72,73]. LPS reaches the cytosol if Gram-negative bacteria evade the phagosome, if LPS is bound by guanylate-binding proteins (GBP) and immunity related GTPases (IRGs) and released from phagosomes, if LPS-containing outer membrane vesicles (OMVs) from extracellular bacteria are taken up by endocytosis, or upon LPS binding to HMGB1 and receptor for advanced glycation endproducts (RAGE) mediated endocytosis [67,68,69,74,75,76,77,78,79,80,81,82,83,84,85].

## 3. Inflammasome Assembly

Inflammasome sensors generally exist in an autoinhibited conformation, with the LRR blocking the NACHT-mediated oligomerization, the AIM2-PYD blocking the HIN-200 domain and 14-3-3 chaperon binding reinforcing the inactive Pyrin conformation. Upon PAMP/DAMP or pathogen activity sensing these blocks are released, which enables the recruitment of the adaptor protein apoptosis-associated speck like protein containing a CARD (ASC), and recruitment and activation of caspase-1 [15,47,48,49,50,86]. ASC encodes a PYD and a CARD and can therefore be recruited to the PYD or CARD of inflammasome sensors through homotypic PYD-PYD or CARD-CARD interactions [87,88,89]. Activated inflammasome sensors oligomerize and recruit ASC, causing an increase in the local ASC concentration and nucleation of ASC polymerization, which then proceeds by a prion-like, self-perpetuating mechanism. Therefore, ASC polymerization is a critical, non-reversible step in initiating inflammasome responses [90,91,92,93]. Subsequently, CARD-CARD mediated recruitment of the effector protease caspase-1 by ASC or by direct binding to the CARD of NLRCs and NLRP1, induces nucleation of caspase-1 polymerization, caspase-1 proteolysis, and induced proximity-mediated auto-activation [94,95,96,97,98,99]. Some variations from this mechanism exist, as neither NLRP1 nor NLRC4 require ASC, but its presence enhances inflammasome responses. Nevertheless, also NLRC4 activation occurs by nucleated polymerization, with a single bacterial PrgJ-activated NAIP2 or flagellin-activated NAIP5, nucleating NLRC4 polymerization. This scaffold, consisting of one NAIP molecule and 10-12 NLRC4 molecules, promotes continuous oligomerization and formation of new nucleation/oligomerization surfaces to further activate NLRC4 and caspase-1 [100,101,102]. NLRP1-mediated caspase-1 activation proceeds by a distinct and unique mechanism. As described above, autoproteolytic cleavage between the two FIIND subdomains, ZU5 and UPA, and proteasomal degradation of the NLRP1 N-terminus, releases the C-terminal UPA-CARD fragment, which self-assembles into a caspase-1 activating scaffold [19]. These aggregates of polymerized inflammasome scaffolds can be observed in cells as a cytosolic speck-like structure ranging from 0.8–1 μm in diameter [103,104]. A major function of caspase-1 is the proteolytic processing and maturation of the pro-inflammatory cytokines pro-IL-1β and pro-IL-18, prompting the release of their bioactive forms [105,106,107]. Both cytokines are lacking a classical signal peptide, but caspase-1 mediated removal of the pro-peptide allows for the enrichment of IL-1β from the cytosol to PIP2-enriched plasma membrane ruffles, likely via cleavage of the endosome autoantigen 1 and a polybasic motif in the cytokines [108,109]. Release occurs from living cells through membrane pores and during a lytic type of inflammatory cell death called pyroptosis [108,109,110,111]. Pyroptosis also leads to the release of polymerized ASC particles, which are phagocytized by neighboring cells and act as DAMP to propagate and further amplify inflammasome responses [103,112,113]. Pyroptosis is induced following proteolytic cleavage of gasdermin-D (GSDMD) at Asp275 in the linker, thereby releasing the N-terminal fragment (GSDMD-N) from the auto-inhibitory C-terminal fragment [114,115]. GSDMD-N then binds to membrane lipids, primarily cardiolipin and phosphatidylinositol, and polymerizes to create membrane pores [116,117,118,119,120,121]. Cardiolipin is also present in the inner mitochondrial membrane, and consequently, GSDMD-N also permeabilizes mitochondrial membranes, leading to release of cytochrome-c and subsequent caspase-3 activation to amplify inflammasome responses [122]. Moreover, cardiolipin is also present on bacterial membranes, which can be directly lysed by GSDMD-N [119]. Pyroptosis has long been considered a cell death pathway directed by caspase-1. However, in response to cytosolic lipopolysaccharide (LPS), the non-canonical inflammasome caspases related to caspase-1, namely human caspase-4 and caspase-5 and mouse caspase-11, also activate pyroptosis, but not cytokine processing [68,69,74,85]. Nevertheless, activation of the non-canonical inflammasome licenses the canonical NLRP3 inflammasome for cytokine release [68,71]. This step involves a caspase-11 and caspase-4-mediated reduction of intracellular K^+^ levels, which is a common canonical NLRP3 inflammasome activating step. Proteolytic cleavage of pannexin-1 by active caspase-11 or caspase-4 mediates ATP release, which activates the ATP-gated P2X7 channel, and promotes ion flux [71,123,124,125]. However, this mechanism has recently been questioned and attributed to NLRP3 activation during apoptosis, but not pyroptosis [126]. In addition, activation of ROS and transient receptor potential channel 1 (TRPC1) may also be involved [127,128].

In addition to pathogen and danger signal-activated inflammasomes, mutations and allelic variations in several inflammasome sensors, including NLRP1, NLRP3, NLRP7, NLRP12, NLRC4, AIM2, and Pyrin, have been implicated in an increasing spectrum of autoinflammatory diseases. Alterations of the sensor sequence can lead to uncontrolled inflammasome activation, which is uncoupled from a PAMP or DAMP, but causes or at least contributes to excessive pyroptosis and cytokine release [129,130,131,132,133,134,135]. Therefore, a firm regulation of inflammasome responses is imperative for preventing detrimental consequences. Identification of new regulatory mechanisms of caspase-1 activation, like the discovery of novel inhibitory molecules, provides an opportunity to apply this knowledge to translational approaches in the clinic. It is now well established that inflammasome responses proceed by a two-step mechanism of “priming” and “activation”, through transcriptional and posttranslational events mediated by specific protein interactions [24,136,137]. In addition, inflammasome components are maintained in an inactive conformation by a platitude of molecular mechanisms, including phosphorylation, ubiquitination, *s*-nitrosylation, sumoylation and a number of binding partners, including NEK7, GBP5, 14-3-3, and others [24,136]. In this review, we summarize the recent developments focusing on a distinct regulatory mechanism of competitive binding, mediated by a family of inflammasome inhibitory proteins, which interfere with the crucial initiating step of nucleated inflammasome assembly, namely the CARD- and PYD-only proteins, COPs, and POPs.

## 4. Inflammasome Regulation by PYD-Only Proteins (POPs) and CARD-Only Proteins (COPs)

**Figure 1:**
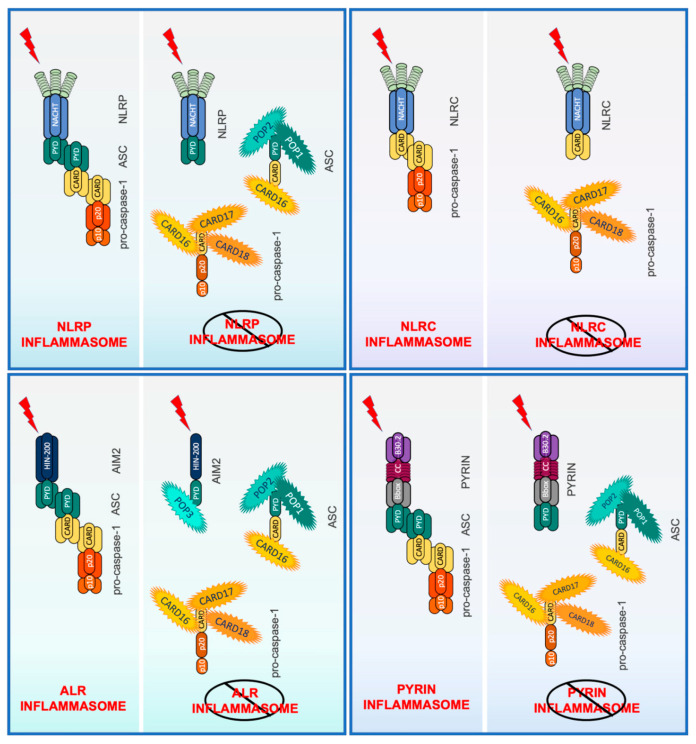
Overview of NLRP, NLRC, Aim2 and pyrin inflammasome regulation by POPs and COPs by interfering with the essential PYD-PYD and CARD-CARD interactions required for inflammasome scaffold assembly and nucleation of ASC and caspase-1 polymerization.

**Figure 2:**
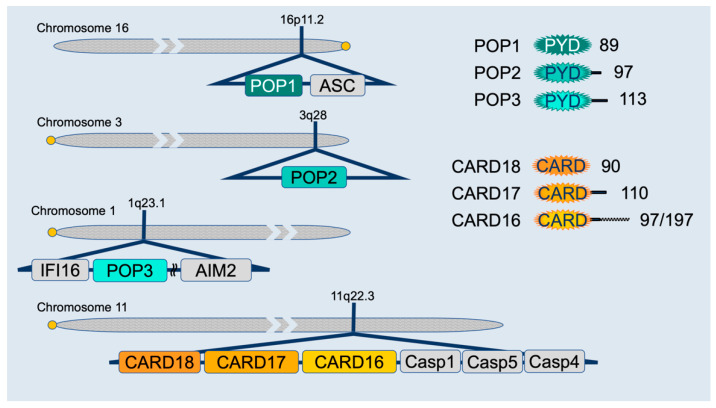
Schemata of POPs and COPs and their chromosomal organization in proximity to key inflammasome components.

Both the PYD and CARD belong to the death domain fold (DDF) superfamily of protein–protein interaction domains, present in proteins regulating cell death and inflammation. They contain a bundle of six anti-parallel α-helices with a charged outer surface and a stabilizing hydrophobic core [138,139,140]. PYDs and CARDs are about 80–90 amino acids long and commonly mediate homotypic interactions, with the charged and hydrophobic surface patches dictating binding specificities. Proteins usually contain a CARD or PYD in combination with other domains to enable complex formation and signaling [89,141]. While CARDs can be on the N- or C-terminus of proteins, PYDs are exclusively present at the N-terminus of proteins, and a PYD or a CARD is present in all inflammasome core proteins, including caspases, NLRs, ALRs, ASC, and Pyrin [138]. However, particularly CARD-containing proteins, are also linked to other signaling pathways, including NF-κB activation. As discussed above, inflammasome assembly relies on PYD- and CARD-mediated protein interactions to nucleate and perpetuate protein polymerization [91,92,93]. Therefore, small endogenous proteins encoding a single PYD or CARD provide a unique, competitive binding mechanism to interfere with PYD and CARD-mediated interactions of multi-domain proteins, and consequently, interfere with assembly and nucleation of inflammasome components (Figure 1). Furthermore, interference with critical interactions reduces the local density of essential inflammasome components and thereby raises the threshold for inflammasome activation. The PYD-only proteins (POPs) POP1 (PYD containing 1, PYDC1), POP2 (PYDC2), and POP3 (PYDC5) can bind to inflammasome sensors or the adaptor ASC, but all three human CARD only proteins (COPs), namely CARD16 (COP/PSEUDO-ICE), CARD17 (INCA), and CARD18 (Iceberg), which share various degrees of similarity to the caspase-1-CARD, can bind to caspase-1 and only CARD16 can also bind to the ASC-CARD. Intriguingly, all COPs and POPs are absent from rat and mouse genomes but are specifically encoded in humans and higher primates [142,143,144]. The genomic localization of POPs is in close proximity to other PYD containing proteins, while COPs localize in close proximity to the inflammatory caspase cluster and likely originated from a series of exon duplication events and retro-transposition mechanisms (Figure 2) [143]. A fourth POP, POP4, is encoded by the human NLRP2P pseudogene, but only consists of 45 amino acids that form the first two α-helices, rather than a complete PYD. Nevertheless, POP4 is able to inhibit the NF-κB pathway, but not inflammasomes [145].

### 4.1. PYD-Only Protein 1

POP1 (PYDC1) was the first discovered POP family member and shares 88% sequence homology and 64% sequence identity with the PYD of ASC, including the distribution of charged amino acids. Its genomic location is in close proximity to ASC on chromosome 16p12, and likely originated from exon duplication [146]. POP1 encodes a 90 amino acid protein, which localizes diffusely inside the cell. Despite the high similarity to the PYD of ASC, POP1 does not retain its self-polymerization potential, but colocalizes and interacts with ASC. This interaction is mediated by a negative electrostatic potential surface patch from the α helices 1 and 4 in the ASC-PYD and a positive electrostatic potential surface patch from the α helices 2 and 3 in POP1 [147]. Mutation of K20, K21 and R41 in POP1 and E13 and D48 in the ASC-PYD disrupts their interaction, which is the same binding mode responsible for the NLRP3-ASC interactions and therefore may explain the disruptive potential of POP1 on inflammasome assembly [147,148]. While overexpression in HEK293 cells indicated a role of POP1 in regulating NF-κB, subsequent studies with endogenous POP1 identified its main function to be the disruption of canonical inflammasome assembly. In agreement, siRNA mediated POP1 silencing in macrophages enhances inflammasome-mediated IL-1β and IL-18 secretion and pyroptosis [149]. Conversely, increasing POP1 expression results in the inhibition of ASC polymerization and caspase-1 activation, and eventually prevents IL-1β and IL-18 secretion. Mechanistically, POP1 binding to ASC prevents sensor-induced nucleation of ASC polymerization, the crucial initial step in inflammasome assembly [149]. Furthermore, POP1 also abolishes the inflammasome-driven release of polymerized ASC particles, which act as danger signals for bystander cells to propagate inflammasome responses and are also prevented by POP1 [149]. The murine ASC-PYD is 88% identical to the human ASC-PYD, and therefore POP1 expression introduced into mouse macrophages was also able to impair inflammasome responses [149]. In vivo, monocyte, macrophage and Dendritic cell (DC)-restricted transgenic POP1 expression from the human CD68 promoter in mice, potently prevents IL-1β-mediated systemic inflammation and lethality in response to LPS administration [149]. Moreover, POP1 transgenic mice are completely protected from CAPS caused by the NLRP3-R260W mutation, as evident from a restored normal development and prevention of lethality, usually driven by systemic inflammation. Hence, POP1 can prevent detrimental consequences of inflammatory disease [149]. This was a crucial finding, as PBMCs from CAPS patients have decreased POP1 transcript levels, when compared with healthy controls and a similar trend was also observed in leucocytes from septic patients. Normally, POP1 expression is upregulated by inflammatory stimuli, including IL-1β and IL-18, indicating that POP1 functions in a regulatory feedback loop to potentially prevent excessive inflammasome activation and to eventually resolve these responses. Hence, reduced POP1 levels in patients may be insufficient to prevent excessive inflammasome responses, which may suggest that a POP1 based therapy could be a novel treatment approach [149]. Indeed, the administration of recombinant, cell penetrating POP1 ameliorates systemic inflammation [149].

### 4.2. PYD-Only Protein 2

POP2 (PYDC2) displays unique as well as overlapping functions with POP1 and encodes a 97 amino acid protein, which shares 68% sequence homology with the NLRP2-PYD, 50% with the NLRP7-PYD and only 37% similarity with the ASC-PYD [150,151]. NLRP7-PYD-based homology modelling suggested α helices 1 and 4 form a negatively charged surface, which may enable interaction with the positive surface patch of NLRP-PYDs and ASC-PYD, but a positively charged surface is absent in POP2. Similar to POP1, also POP2 expression is upregulated by inflammatory stimuli, and POP2 also co-localizes with ASC and regulates inflammasome assembly and downstream responses, but regulates also activation of NF-κB [150,151,152,153,154]. These two activities can be separated, as α helix 1 is crucial for regulating NF-κB, while the acidic residues E6, D8 and E16 are only essential for inhibiting canonical inflammasomes, but are not required for NF-κB inhibition [152]. Based on binding to ASC, POP2 also broadly blocks canonical inflammasome responses, including caspase-1 activation, pyroptosis, and release of IL-1β and IL-18 [150,151,152,153,154]. Similar to POP1, also POP2 mediates the inflammasome inhibitory effect by preventing the nucleation of ASC polymerization, thereby abrogating inflammasome assembly [154]. However, in addition to solely regulating inflammasome assembly, POP2 also regulates the priming step by regulating TLR-mediated NF-κB activation. While the exact mechanism remains elusive, dampening the non-canonical IKKε and IκBα have been implicated [150,153,154]. Also, these pathways are sufficiently conserved between mice and humans, and therefore transgenic expression from the human CD68 promoter, thereby restricting its expression to monocytes, macrophages, and DCs, or elegantly utilizing its endogenous human promoter, phenocopies its function in mice [153,154]. Canonical inflammasome-driven systemic inflammation is also ameliorated in vivo [153,154]. Furthermore, POP2 transgenic mice were protected from *Francisella tularensis* and *Streptococcus pneumoniae* infection, but not from infection with *Salmonella typhimurium*, which is sensed by the NLRC4 inflammasome independently of ASC [153]. POP2 transgenic mice demonstrated reduced inflammatory cell infiltration and tissue damage, when compared to littermate controls [153]. Mice show elevated levels of interferon (IFN)-γ post infection, due to an increased number of IFN-γ-secreting macrophages promoting homeostasis between inflammatory cytokine production and IFN-γ mediated host defense [153]. Hence, POP2 regulates both, inflammasome priming and activation, while also promoting IFN-γ mediated antibacterial activities. Consequently, POP2 may promote host defense without the negative consequences resulting from excessive inflammation.

### 4.3. PYD-Only Protein 3

POP3 (PYDC5) is localized within a type I IFN-inducible genomic cluster encoding all four members of the human PYHIN (HIN-200) family between IF16 and PYHIN1 on chromosome 1q23, which are all characterized by a PYD and one or two C-terminal oligonucleotide binding HIN-200 domain(s), except for POP3, which only encodes a PYD [144,155,156]. POP3 encodes a 113 amino acid protein of five α helices, as demonstrated by homology modeling, diverging from other death domain folds. It only shares 19% sequence identity to ASC, but is 61% identical to AIM2, also suggesting origination by exon duplication. Indeed, POP3 interacts with the PYD of AIM2 and IFI16, but unlike POP1 and POP2, does not bind to the ASC-PYD. However, comparable to other POPs, POP3 interaction with AIM2 also abolishes the AIM2-ASC complex formation and the resulting nucleation of ASC polymerization [144]. Rather than binding to the adaptor, POP3 directly binds to the sensor, which also results in a much more restricted function, as POP3 specifically impairs the cytosolic dsDNA-induced inflammasome response without affecting other canonical inflammasomes [144]. POP3, through interactions with AIM2 and IFI16, blocks inflammasome responses induced by infection with dsDNA viruses, including Vaccinia virus (AIM2), CMV (AIM2), and KSHV (IFI16) [144]. Similar to POP1 and POP2, the function of POP3 is also sufficiently conserved and human POP3 binds to mouse AIM2 and IFI16 (p204) [144]. Therefore, transgenic mice with monocyte, macrophage and conventional DC-specific POP3 expression, revealed impaired AIM2-mediated antiviral defense through IL-18-mediated IFN-γ production in response to MCMV infection, resulting in higher splenic viral titer [144]. However, as expected from cell-based studies, POP3 transgenic mice did not reveal any altered response to MSU crystal injection, which induces an NLRP3 inflammasome-mediated response [144]. Viral infections are potent inducers of type I IFNs, which positively regulate POP3 transcription and protein stability [144]. Therefore, it is quite feasible that POP3 is located within a type I interferon-induced inflammasome feedback loop to eventually resolve cytosolic DNA driven inflammasome responses.

### 4.4. CARD16 (COP/Pseudo-ICE)

There are two isoforms of CARD16, a long and a short isoform, though only the short variant has been studied. The short isoform contains a 91-amino acid CARD and six additional amino acids at the carboxy terminus and the long isoform contains the CARD and 106 additional amino acids at the carboxy terminus [157,158]. CARDs are known for their ability to oligomerize and to interact with other CARDs. Accordingly, CARD16 is able to self-oligomerize, similar to the CARD of caspase-1, forming filament-like structures. It also shares 97% amino acid sequence similarity with, and is able to bind to the CARD of caspase-1 [157,158,159]. Interestingly, mutation of D27 or R45 within CARD16 to G27 or C45, which are the correlating amino acids within CARD17, resulted in a non-filament forming form of CARD16. Furthermore, CARD16 interacts with the CARD of ASC, co-localizing in perinuclear ASC-specks [159]. Taken together, this suggests that CARD16 could regulate inflammasome assembly, caspase-1 activation, and cytokine maturation. Indeed, CARD16 was able to function as an inhibitor for IL-1β maturation and secretion, but was also able to induce IL-1β secretion through its ability to bind, aggregate and activate caspase-1, since the non-filament forming mutants of CARD16 were unable to induce IL-1β secretion to the same extent as wildtype CARD16 [157,158,159]. While inflammasome activation was observed in epithelial Hela cells, inflammasome inhibition was detected in monocytic THP-1 cells [158,159]. Hence, the expression of other co-regulators in different cell types might be able to alter the effects of CARD16 on caspase-1 activation or may depend on CARD16 expression levels. Further evidence for an inhibitory function of CARD16 on caspase-1 was discovered when RIP2 mediated caspase-1 activation and IL-1β secretion was blocked by CARD16 [157]. Numerous CARD-containing proteins have been implicated to play a role in the NF-κB signaling pathway, and at least by overexpression in HEK293 cells, CARD16 was able to activate NF-κB [158]. However, further studies will be necessary to better understand the physiological role of CARD16 on inflammasome regulation and NF-κB signaling. Interestingly, among all CARD only proteins, CARD16 shares the highest (83.8%) sequence homology with the caspase-1 promoter region, and therefore CARD16 expression is comparable to caspase-1 expression in lymph nodes, placenta, spleen, bone marrow, PBMCs, and macrophages [158,159].

### 4.5. CARD17 (INCA)

CARD17 encodes a 110 amino acid protein that is 81% identical to the caspase-1 CARD [160]. Although self-oligomerization is a very common feature of CARDs, CARD17 is unable to self-oligomerize upon expression in Hela cells. However, reminiscent to CARD16, also CARD17 is able to bind to the CARD of caspase-1 and interferes with the self-oligomerization of the caspase-1 CARD, suggesting that CARD17 could have an inhibitory effect on caspase-1 activation by blocking caspase-1 self-oligomerization. Indeed, CARD17 is able to block IL-1β maturation and secretion. While CARD16 binds to caspase-1 and ASC, CARD17 did not interact with the ASC-CARD and did not co-localize with ASC in specks [159,160]. However, CARD17 was able to interfere with caspase-1 and ASC co-localization in specks and caused disperse caspase-1 localization [159,160]. Contrary to CARD16, CARD17 does not affect RIP2-mediated NF-κB activation. Electron microscopy revealed that CARD17 localizes to the tip of caspase-1 oligomeric filaments to cap filaments and prevent further filament elongation, due to the lack of two of the six complementary binding surfaces necessary for nucleating polymerization [161]. Interestingly, CARD17 does not form a stable complex with the CARD of caspase-1 to sequester the monomeric form but rather caps the oligomeric form [161]. Similar to CARD16, also the upstream promoter regions of CARD17 and Caspase-1 share 80.2% sequence homology [159]. However, in spite of the strong promoter homology, the expression profile of CARD17 and caspase-1 differ, except for the positive regulation by IFN-γ, suggesting a unique regulation [160].

### 4.6. CARD18 (ICEBERG)

CARD18 was the first identified COP and encodes a 90 amino acid protein with only 52% homology to the CARD of caspase-1 [158,162]. Its NMR structure has been solved, revealing that α helices, 1, 4, and 6 are comprised of positively charged residues and α helices 2 and 5 are comprised of negatively charged residues comparable to caspase-1. Hence, electrostatic interaction between CARD18 and caspase-1 should be possible [94,162,163]. Indeed, CARD18 binds to caspase-1 by CARD-CARD interaction and inhibits caspase-1 oligomerization and activation [158,162]. However, a recent report suggests that CARD18 actually promotes caspase-1 polymerization and filament formation, thereby activating caspase-1, but without promoting additional IL-1β release [161]. However, expression of CARD18 consistently reduces IL-1β maturation and release in other studies [158,162]. Expression of CARD18 is quite distinct from other COPs, as it is not affected by IFN-γ but enhanced by LPS and TNF [158,162]. However, more efforts are necessary to clarify the controversial role of CARD18 in regulating inflammasome activation. Interestingly, CARD18 expression can be detected in keratinocytes and is significantly increased in response to inflammatory stimuli. Silencing of CARD18 also decreases the expression of inflammasome components but increases IL-1β secretion in response to inflammatory stimuli, indicating that CARD18 negatively regulates inflammasome activation. Strikingly, the expression of CARD18 is altered in psoriasis, which may contribute to disease pathogenesis [164].

## 5. Conclusions

Tremendous progress has been made in delineating the molecular mechanisms responsible for inflammasome activation, assembly, and the downstream responses, as well as identification of numerous inflammasome activators and regulators. Nucleated polymerization of PYD and CARD-containing inflammasome components emerged as a key step in inflammasome assembly. Hence, the interference of POPs and COPs with this critical step provides a regulatory mechanism for fine tuning inflammasome assembly and activation. In spite of recent advances, COPs and POPs are still largely understudied, and further work is needed to provide deeper insights into the precise contribution of each individual family member and their unique and shared aspects to control inappropriate inflammasome responses. There is evidence that COPs and POPs are lacking in inflammatory diseases. For instance, CAPS and sepsis patients have significantly less POP1 expression in whole blood than healthy controls [149], POP1, POP2, and CARD18 are downregulated in periodontal disease [165] and CARD18 is highly expressed in the epidermis, but expression is lost in lichen planus [166]. Hence, we expect that there is a unique expression pattern of POPs and COPs in different tissues and cell types, either during homeostasis or during inflammatory immune responses. Since the overactivation of inflammasomes is directly associated with an increasing spectrum of inflammatory disorders, we believe that the expression of COPs and POPs is critical to provide a balanced and timely immune response and to maintain homeostasis.

## Abbreviations

AIM2	absent in melanoma 2
ASC	adaptor protein apoptosis-associated speck like protein containing a CARD
ASP	aspartic acid
ATP	adenosine triphosphate
CAPS	cryopyrin-associated periodic syndrome
CARD	caspase recruitment domain
CMV	cytomegalovirus
COP	caspase recruitment domain-only protei
DAMP	damage associated molecular pattern
DDF	death domain fold
DC	Dendritic cell
dsDNA	double stranded deoxyribonucleic acid
FIIND	function to find domain
GBP	guanylate-binding protein
GSDMD	gasdermin D
HIN-200	hematopoietic interferon-inducible nuclear proteins with a 200-amino-acid repeat
HMGB1	High Mobility Group Box 1
IFN	interferon
IκB	inhibitor of nuclear factor-κB
IKK	inhibitor of nuclear factor-κB (IκB) kinase
IL	interleukin
IRG	immunity related GTPase
KSHV	Kaposi Sarcoma-Associated Herpesvirus
LPS	lipopolysaccharide
LTA	lipoteichoic acid
MAPK	mitogen-activated protein kinase
MCMV	murine cytomegalovirus
NACHT	NAIP, CIITA, HET-E and TEP1
NAIP	apoptosis inhibitory NLR protein
NF-κB	Nuclear Factor kappa-light-chain-enhancer of activated B cells
NLR	Nod like receptor
NLRC	NLR caspase recruitment domain-containing
NLRP	Nod like receptor (NLR) family pyrin domain-containing
OMV	outer membrane vesicle
PAMP	pathogen associated molecular pattern
PBMC	peripheral blood mononuclear cell
POP	PYRIN domain-only protein
PRR	pattern recognition receptor
PYD	PYRIN domain
PYDC	PYRIN domain containing
Ser	serine
T3SS	type III secretion system
TLR	Toll-like receptor
TNF	tumor necrosis factor
TRPC1	transient receptor potential channel 1
